# Optimization of Culture Conditions for Enhanced Growth, Lipid and Docosahexaenoic Acid (DHA) Production of *Aurantiochytrium* SW1 by Response Surface Methodology

**DOI:** 10.1038/s41598-018-27309-0

**Published:** 2018-06-11

**Authors:** Yusuf Nazir, Shuwahida Shuib, Mohd Sahaid Kalil, Yuanda Song, Aidil Abdul Hamid

**Affiliations:** 1 School of Biosciences and Biotechnology, Faculty of Science and Technology, Universiti Kebangsaan Malaysia, Selangor, Malaysia; 2 Department of Chemical and Process Engineering, Faculty of Engineering and Built Environment, Universiti Kebangsaan Malaysia, Selangor, Malaysia; 3 Colin Ratledge Center for Microbial Lipids, School of Agriculture Engineering and Food Science, Shandong University of Technology, Zibo, 255049 China

## Abstract

In this study, optimization of growth, lipid and DHA production of *Aurantiochytrium* SW1 was carried out using response surface methodology (RSM) in optimizing initial fructose concentration, agitation speed and monosodium glutamate (MSG) concentration. Central composite design was applied as the experimental design and analysis of variance (ANOVA) was used to analyze the data. ANOVA analysis revealed that the process which adequately represented by quadratic model was significant (p < 0.0001) for all the response. All the three factors were significant (p < 0.005) in influencing the biomass and lipid data while only two factors (agitation speed and MSG) gave significant effect on DHA production (p < 0.005). The estimated optimal conditions for enhanced growth, lipid and DHA production were 70 g/L fructose, 250 rpm agitation speed and 10 g/L MSG. Consequently, the quadratic model was validated by applying the estimated optimum conditions, which confirmed the model validity where 19.0 g/L biomass, 9.13 g/L lipid and 4.75 g/L of DHA were produced. The growth, lipid and DHA were 28, 36 and 35% respectively higher than that produced in the original medium prior to optimization.

## Introduction

The importance of omega 3 (ω-3) and omega 6 (ω-6) polyunsaturated fatty acids (PUFAs) such as gamma linolenic acids (GLA), arachidonic acid (ARA), eicosapentaenoic acid (EPA), docosahexaenoic acid (DHA) has long been discovered and discussed^1^. PUFA such as ω-3 fatty acid normally contains heterocyclic compounds with two or more double bonds, of which the last double bond is located at the third carbon atom from the methyl terminal. Docosahexaenoic acid (DHA, 22:6) is a particularly important ω-3 PUFA, with a 22-carbon chain and six double bonds^2^.

Since late 1980s, it became clear that DHA plays a significant role in upholding human health especially in infants^3^. DHA is naturally found in breast milk and it is essential for the development of brain and eyes of an infant^4^. Furthermore, DHA is also proven for its effectiveness in curing several diseases, such as cancer, coronary heart disease, hypertension, depression, type-2 diabetes mellitus, atherosclerosis, and thrombosis^5–7^. Thus, DHA is widely used as a nutraceutical component in the food and feed market^8^. Currently, the commercial source of DHA is fish oil derived from cold water fatty fish such as Tuna and Salmon. However, several drawbacks have been found for fish oils such as the presence of highly saturated fatty acids, contamination by hazardous substances, fishy odor, low quantity of DHA, and complications in purification processes^9,10^. Therefore, an alternative DHA source to fish oils were explored.

Microbial oil, being produced in a controlled environment, is one of the current topics of massive research because it has many advantages compared to fish oil^11^. It is noteworthy that fishes obtain ω-3 fatty acids from zooplankton which feed on *Thraustochytrids*. Hence, current studies are diverted on producing PUFA directly from this microorganism. Two commercially available marine *Thraustochytrids* which are *Aurantiochytrium/Schizochytrium* and *Crypthecodinium cohnii* have been shown to be an excellent DHA producers. Members of the genus *Aurantiochytrium*, as well as other strains of *Thraustochytrids*, are able to produce large amounts of oil, which is up to 60% of the biomass and DHA can comprise as much as 35–55% of the total fatty acids (TFAs)^12^. DHA from *Aurantiochytrium* has also been proven to be safe for human consumption and it is free from the common algal toxins such as domoic acid and prymnesin produced by some members of its kingdom, Chromista^13^.

Due to its benefits and advantages over fish oil, many efforts have been made to increase the DHA productions of these *Thraustochytrids* and to make it industrially feasible. Of the various efforts, optimization of culture conditions has been proven to significantly improved growth and DHA production by most *Thraustochytrids* including *Aurantiochytrium* sp^14,15^. Thus, this study intends to optimize the culture condition to enhance the growth, lipid and DHA production of our *Thraustochytrids* isolate. There are two different aspects that we considered in optimizing the culture condition; (1) optimization of medium components and (2) optimization of environmental parameters such as agitation speed and temperature of incubation. In optimizing the medium components, carbon and nitrogen content are the two most important components to be considered as their role in lipid accumulation has been extensively established.

According to Ratledge *et al*.^3^, lipid accumulation in oleaginous microbes is triggered by nutrient imbalance in the culture medium. When nitrogen sources are depleted, excess carbon in the medium continues to be assimilated by the cells and converted into storage lipids. Besides, environmental aspects of the cultivation such as the agitation speed also play a significant role in enhancing the amount of lipid accumulated in the cells. Therefore, it is important to know the suitable and optimal agitation speed for the *Thraustochytrids* cultivation so that the dissolved oxygen content in the medium could be improved and cell breakage due to sheer effect could be avoided which consequently results in enhanced growth of the cell^16^. Hence, optimizing the carbon, nitrogen and agitation speed are key factors to achieve high cell growth as well as enhanced lipid and DHA production by *Auratiochytrium* sp.

Therefore, the aim of this study is to optimize fructose and MSG which are the major carbon and nitrogen source in the cultivation media as well as agitation speed to enhance growth, lipid and DHA production of locally isolated *Auranthiochytrium* SW1 by using RSM. Fructose was used instead of glucose and other carbon sources is in conjunction from our previous experiment which concluded that fructose is the best carbon sources for growth, lipid and DHA production by this *Thraustochytrids* strain^17^.

## Results and Discussions

### Profiles of growth, lipid and DHA content of *Aurantiochytrium* SW1 in unoptimized production medium

**Figure 1:**
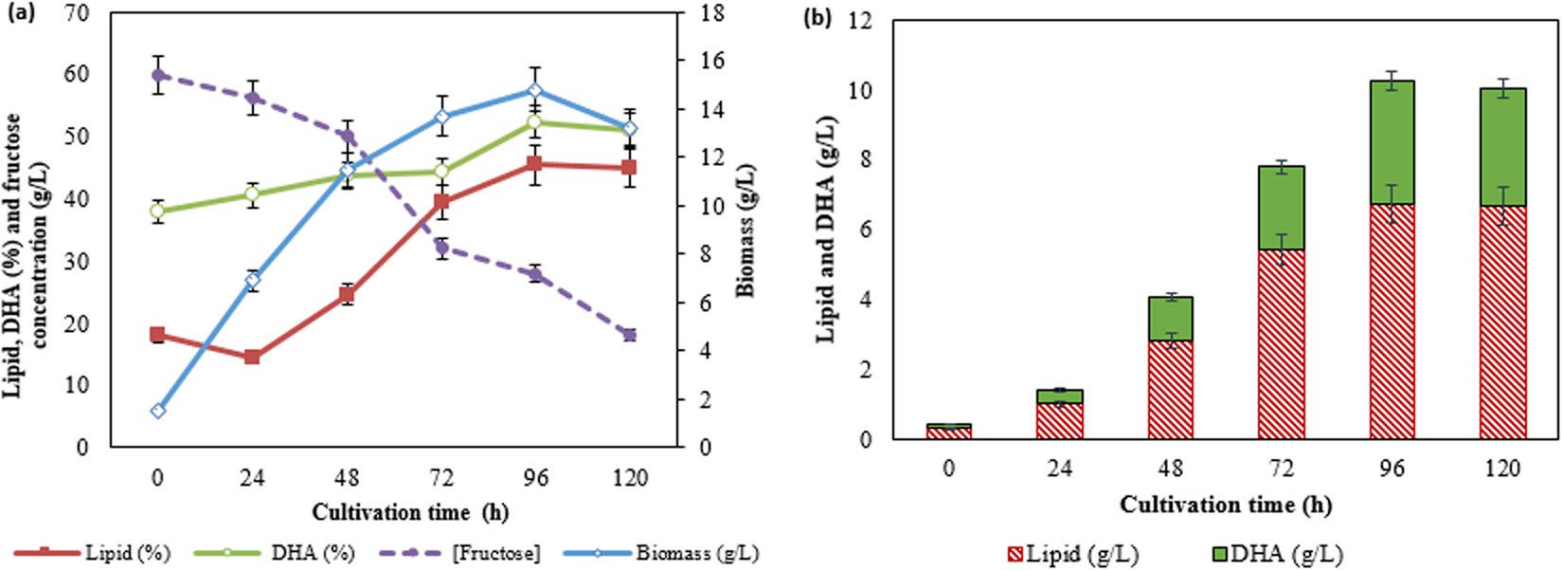
(**a**) Growth, lipid content (%), DHA content (%) and fructose assimilation as well as (**b**) lipid and DHA yield (g/L) of *Aurantiochytrium* SW1 grown in 500 mL shake flask at 30 °C, agitation speed of 250 rpm for 120 h.

The profiles of biomass, lipid, DHA, as well as fructose assimilation of *Aurantiochytrium* SW1 cultured in 500 mL flask containing 100 mL of medium, was demonstrated in Fig. 1a and b. *Aurantiochytrium* SW1 (would be addressed as SW1 henceforth) has a typical growth profile as other *Thraustochytrids* as reported by Song *et al*.^18^ and Goa *et al*.^19^, where the cell growth (biomass) increased rapidly up to 48 h and then slowed down until 96 h of cultivation. The cells begin to enter death phase beyond 96 h of cultivations. During the rapid growth phase, biomass production increased significantly from 1.5 to 11.0 g/L with the specific growth rate of 0.19 g/L/h. This is likely due to lack of competition among SW1 cells since there are still abundance of nutrients and adequate space in the culture medium. The maximum biomass concentration achieved was 14.8 g/L at 96 h of cultivation. SW1 also shown to have a short lag phase and not observable after 24 h of cultivation (Fig. 1a). Short lag phase in microbial growth is highly accentuated to achieve high productivity, especially for industrial-scale production.

The lipid accumulation in SW1 was slow at the initial stage of cultivation (0 to 24 h) and started accumulated substantially from 24 h until late stationary phase (96 h) where the lipid content increased significantly from 12 to 44% of the biomass (g/g biomass) (Fig. 1a). Significant increment in lipid yield (g/L) was also observed with the maximum lipid (6.7 g/L) produced at 96 h of cultivation (Fig. 1b). The pattern of lipid accumulation in SW1 was also shown to be parallel to the biomass production. This result suggested that lipid accumulation in SW1 was growth-associated as it was not initiated by nitrogen limitation. However, this type of lipid accumulation pattern differs from most oleaginous fungi where nitrogen limitation in culture media is mandatory for the onset of substantial lipid accumulation^3^. The lipid accumulation pattern of SW1 was also similar to other heterotrophic algae such as *Crypthecodinium cohnii*, *Schizochytrium* sp. S31 and other *thraustochytrids* sp^20–22^. However, the cause of lipid turnover observed between 96 h to 120 h (Fig. 1a) was not obvious since the carbon source was still abundant in the culture media. Lipid turnover commonly happened in oleaginous microorganisms after exhaustion of carbon sources where the reserved lipid was used for growth and biomass production through β-oxidations process^23,24^.

Further analysis of the lipid was carried out to determine the DHA content of the total fatty acids (TFAs). The result showed that the DHA content was less than 40% at the beginning of the experiment, but it continued to increase as the cultivation progressed achieving 52.3% (3.5 g/L) at 96 h of cultivation (Fig. 1a and b). A similar pattern of DHA production was also reported in *Schizochytrium* sp. S31 and *Aurantiochytrium* sp. YLH70 which were cultured using glycerol and high-fructose corn syrup (HFCS) as the carbon sources respectively^22,25^. The increment in DHA content along with the increase of cultivation time might be associated with the decline in the dissolved oxygen content in the cultivating medium. It was reported that there were two mechanisms involves in fatty acid synthesis in *Thraustochytrids* which are oxygen-dependent fatty acid synthase (FAS) and oxygen independent polyketide synthases (PKS)^26^. FAS most probably involves in synthesizing saturated FAs (C14:0 and C16:0) whereas PKS is responsible to synthesize PUFA, including DHA. As the cultivation period progressed, the content of dissolved oxygen in the culture medium decreases which led to inactivation of the oxygen-dependent enzymes such as desaturases in FAS system. Therefore, the PKS system that does not require dissolved oxygen during the cultivation become dominant and consequently increase the DHA yield and content^22^. However, since the experiment was carried out in shake flask, the dissolved oxygen content was not measured.

Nevertheless, the growth and DHA production by SW1 are still low compared to commercial *Thraustochytrids* strain such as *Aurantiochytrium* sp. KRS101^27^. Therefore, further experiments were carried out to enhance the growth and DHA production via response surface methodology (RSM). RSM is a useful statistical optimization design that has successfully been used in many biological and chemical processes^28,29^. RSM creates an experimental design with the minimum number of experiments and generates a model that can predict the interaction and correlation between a set of independent variables and observed results, thus providing optimized conditions which is differ from the conventional approach of optimization which only investigates one factor at a time^30^.

### Optimization of growth, lipid and DHA production using RSM

In this study, three factors which are concentration of fructose (A), agitation speed (B) and concentration of MSG (C) were optimized to enhance growth, lipid and DHA production of SW1. Optimization process was conducted using RSM based on a central composite design (RSM-CCD). Twenty sets of experiments at a different combination of factors were performed and the mean result of three replicates for biomass, lipid and DHA production (experimental and predicted) were presented (Table 1).

**Table 1:** Design matrix of medium optimization for biomass, lipid and DHA productions.

Std. order	Variables			Results					
	A (g/L)	B (rpm)	C (g/L)	Experimental Values			Predicted Values		
				Biomass (g/L)	Lipid (g/L)	DHA (%)	Biomass (g/L)	Lipid (g/L)	DHA (%)
1	20.00	100.00	2.00	6.72	1.90	36.20	6.52	1.94	36.18
2	100.00	100.00	2.00	9.20	2.42	37.98	9.66	2.57	36.09
3	20.00	300.00	2.00	10.50	2.67	43.39	9.63	2.10	40.31
4	100.00	300.00	2.00	17.36	6.90	42.44	17.29	6.67	41.86
5	20.00	100.00	16.00	6.62	2.44	48.47	6.35	2.27	47.26
6	100.00	100.00	16.00	8.48	2.88	47.81	9.01	3.04	49.10
7	20.00	300.00	16.00	14.12	4.62	53.00	13.32	4.07	53.11
8	100.00	300.00	16.00	20.62	9.23	58.36	20.49	8.79	56.59
9	0.00	200.00	9.00	5.12	0.98	46.00	6.00	1.63	47.93
10	127.27	200.00	9.00	18.50	5.02	49.77	17.93	5.07	50.76
11	60.00	0.00	9.00	1.90	0.15	32.75	0.42	0.00	32.77
12	60.00	400.00	9.00	12.16	4.13	40.44	12.92	4.81	42.21
13	60.00	200.00	0.00	13.14	5.38	35.48	13.50	5.63	39.11
14	60.00	200.00	20.77	14.06	6.76	56.93	14.33	7.18	57.33
15	60.00	200.00	9.00	16.14	8.00	48.65	16.49	6.77	48.71
16	60.00	200.00	9.00	16.8	6.44	49.00	16.49	6.77	48.71
17	60.00	200.00	9.00	16.00	6.05	49.88	16.49	6.77	48.71
18	60.00	200.00	9.00	16.78	6.83	51.00	16.49	6.77	48.71
19	60.00	200.00	9.00	17.20	7.21	46.22	16.49	6.77	48.71
20	60.00	200.00	9.00	16.30	6.23	49.12	16.49	6.77	48.71
A = Fructose concentration				B = Agitation speed			C = MSG concentration		

From the designed matrix, it showed that the experimental biomass, lipid and DHA production are comparable to the predicted values, indicating that the design is suitable for optimizations.

### Optimization of Growth

Optimization of the growth (biomass) was first carried out by determining the pattern of data distribution (Supplementary File 1). The plot shows that the dataset follows a normal distribution indicating the data is appropriate for statistical optimization.

Five models which are mean, linear, 2FI, quadratic, cubic were tested and among all, quadratic model was chosen to analyze the data as it has the highest order polynomial with a significant sum of squares, insignificant Lack-of-Fit and highest *R*^2^. Analysis of Variant (ANOVA) for the response surface quadratic model was conducted to obtain the significance level of the fitted model and the factors affecting the cell growth. Table 2 showed the ANOVA for the quadratic model.

**Table 2:** ANOVA for biomass production by SW1.

Sources	Sum of Squares	DF	Mean Square	F Value	Prob > F
Model	492.97	9	54.77	119.58	<0.0001
A	98.20	1	98.20	214.38	<0.0001
B	240.65	1	240.65	525.35	<0.0001
C	15.83	1	15.83	34.55	0.0002
A^2^	29.76	1	29.76	64.96	<0.0001
B^2^	133.03	1	133.03	290.41	<0.0001
C^2^	16.70	1	16.70	36.46	0.0001
AB	8.82	1	8.82	19.25	0.0014
AC	1.66	1	1.66	3.62	0.0864
BC	4.99	1	4.99	10.90	0.0080
Residual	4.58	10	0.46		
Lack of Fit	3.79	5	0.76	4.76	0.0559
Pure Error	0.79	5	0.16		
Cor Total	497.55	19			
Std. Dev.	0.79		R^2^	0.98	
Adeq. Precision	36.07		Adj R^2^	0.97	

p < 0.005 is significant.

The significance of each coefficient was indicated by the F and *p* values, which also demonstrate the interaction strength between each independent variable. Values of ‘Prob > F’ less than 0.005 indicate that the model is significant and vice versa. Based on Table 2, the p-value of the model was (p < 0.001) and the F-value was large (119.58) which implied that the model was significant to interprate the data. “Lack of Fit p-value” was 0.0559 indicating the Lack of Fit was not significant relative to the pure error. Adequate precision value, which measures signals to noise ratio recorded a preferable value of 36.07. This implied that this model gave sufficient signals required to navigate the design space. A ratio greater than 4 is desirable. The R^2^ value of 0.98 was a good indicator, showing that a high proportion of variability which up to 98% was explained by the data.

**Figure 2:**
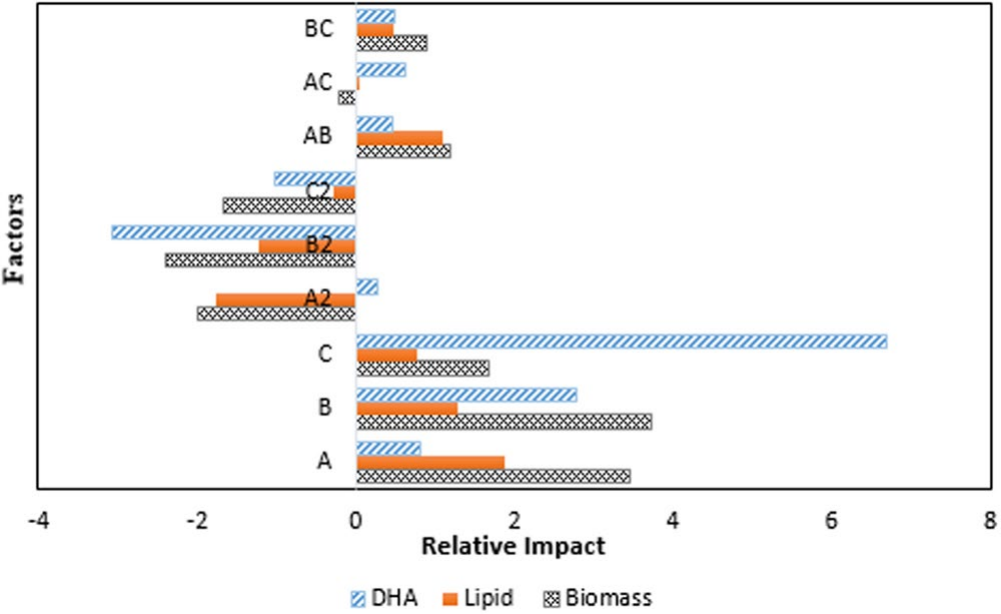
Relative impact of factors on biomass, lipid and DHA production by SW1.

Based on the ANOVA analysis, all the three factors showed significant positive impact on the growth of SW1. The interaction between fructose and agitation speed (AB) as well as agitation speed and MSG (BC) were also significant in enhancing the growth of SW1. According to Anderson and Whitcomb^31^, the coefficients obtained in the final equation can be directly compared to assess the relative impact of factors. Figure 2 illustrated the relative impact of factors affecting the biomass, lipid and DHA production by SW1 via a bar graph. This figure showed that, the influence of each factor for all the response varied among each other and it was concluded that agitation speed had the most significant impact on biomass productions, whereby fructose and MSG had pronounced impact for lipid and DHA production respectively.

Thus, the experimental values obtained from the central composite design (CCD) were regressed using a quadratic polynomial equation, and the regression equation, expressed in terms of the actual factors is shown below.

1 $$\begin{array}{rcl}{\rm{Biomass}}\,({\rm{g}}/{\rm{L}}) & = & -\,5.39809+0.11888\,({\rm{Fructose}})+0.10967\,({\rm{Agitation}})\\  &  & +\,0.30728\,({\rm{MSG}})-9.76324{\rm{E}}004\,({{\rm{Fructose}}}^{2})\\  &  & -\,2.39884{\rm{E}}-004\,({{\rm{Agitation}}}^{2})-0.025894\,({{\rm{MSG}}}^{2})\\  &  & +\,2.62500{\rm{E}}-004\,({\rm{Fructose}}\times {\rm{Agitation}})\\  &  & +\,1.62500{\rm{E}}-003\,({\rm{Fructose}}\times {\rm{MSG}})\\  &  & +\,1.12857{\rm{E}}-003\,({\rm{Agitation}}\times {\rm{MSG}})\end{array}$$

#### Effects of the variables on the growth of SW1

**Figure 3:**
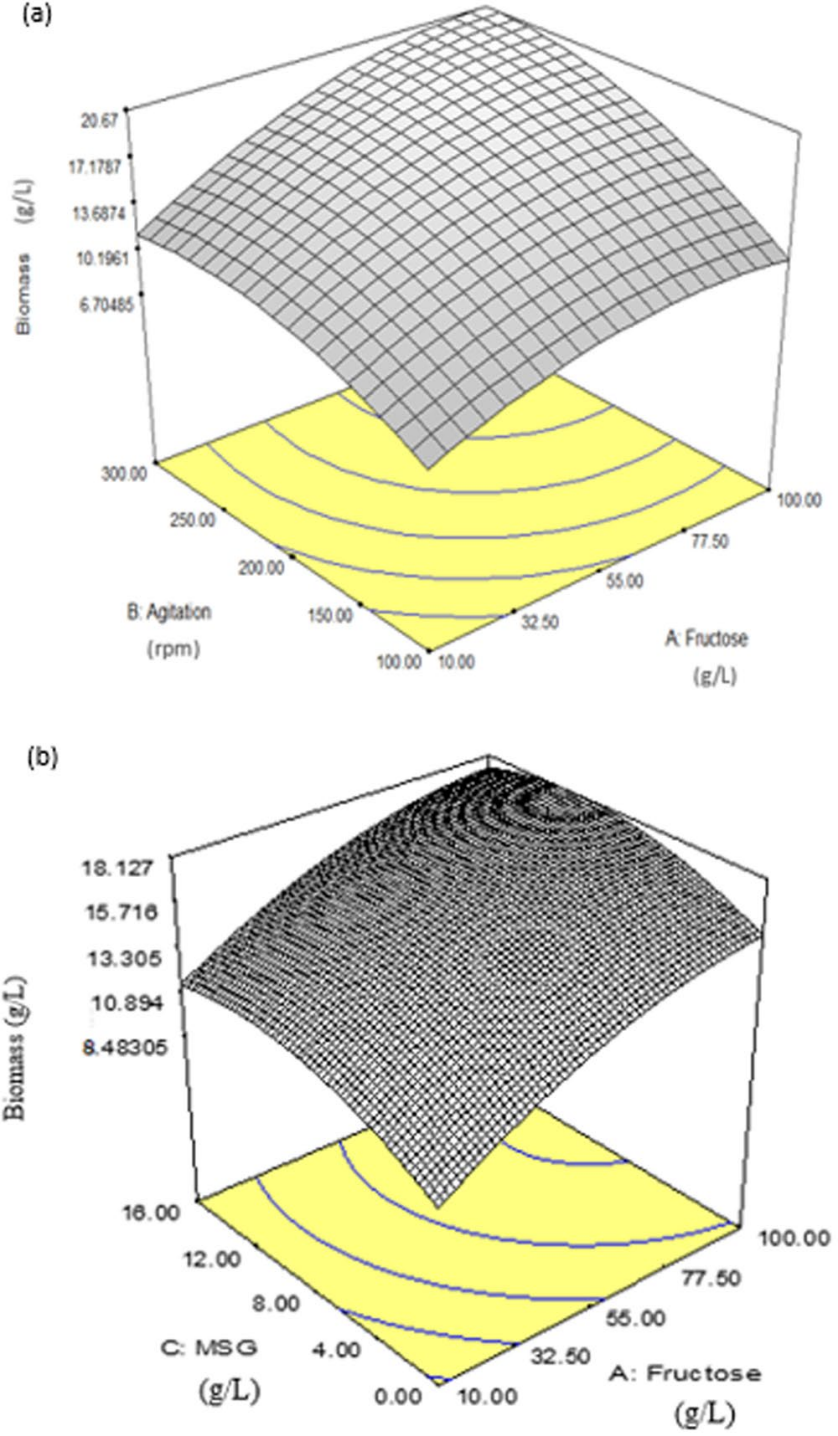
(**a**) Interaction of fructose concentration and agitation speed (AB) as well as (**b**) Interaction between fructose and MSG concentration (AC) for biomass production of SW1.

The three-dimensional (3D) response surface was plotted to study the interaction of the three factors on the growth of SW1 (Fig. 3a and b). This type of graphical visualization allows the relationships between the experimental levels of each factor, the response and the type of interactions between test variables, which is necessary to establish the optimal medium components and culture conditions. Figure 3(a) showed the 3D plot of interaction between fructose and agitation speed (AB) on biomass production of SW1.

The interaction of these two factors (Fig. 3a) gave significant impact on biomass production with the P < 0.005 (Table 2). Maximum biomass was produced when both factors were at maximum level (100 g/L and 300 rpm) respectively and vice versa (Fig. 3a). This could be explained that carbon sources (which referred to fructose) is an essential macro nutrient and plays an important role as a source of energy, building block for DNA, protein, lipid and some metabolites while agitation is essential to increase the dissolved oxygen in the medium where oxygen is vital for the growth of *Aurantiochytrium* sp^32^. When these two factors are at a minimum level, carbon and dissolved oxygen content are also minimal which consequently gave negative impact on the growth of SW1.

However, the individual impact of each factor was shown to have a more extensive influence on biomass production of SW1 with the estimated coefficient of 3.21 and 3.37 for fructose and agitation speed respectively, higher than when both factors interacted (1.27) (Fig. 2). The individual effect of the agitation speed on biomass productions was clearly perceptible when SW1 was cultured at different agitation speed while other factors were constant as shown in run (1 and 3), as well as (5 and 7) (Table 1). Based on Fig. 3(a), the optimal agitation speed for biomass production is 250 rpm and cultivating SW1 beyond this value affects the biomass production negatively. Similar results have been reported by Hong *et al*.^33^ which showed that cultivating *Aurantiochytrium* sp. KRS101 at high agitation speed resulted in reduction of biomass production.

Furthermore, independent impact of fructose on biomass production showed that 70 g/L was the optimal concentration and increasing the fructose concentration beyond this value resulted in inhibition of the growth of SW1 (Fig. 3a). This result is in agreement with the study conducted by Wong *et al*.^34^ and Yokochi *et al*.^35^ which reported that the best concentration of carbon sources for growth and DHA production by *Thraustochytrid* sp is within the range of 60–100 g/L, and cultivating *Thraustochytrids* with carbon sources over 100 g/L resulted in inhibition on growth of the cell. However, a study conducted by Yu *et al*.^25^ reported that *Aurantiochytrium* sp. YLH70 was able to utilize over 150 g/L HFCS after 114 h of cultivation, indicating the capability of substrate consumption differ among the *Thraustochytrid* sp.

The interaction between fructose and MSG (AC) in biomass production of SW1 (Fig. 3b) is also important. Highest biomass was achieved at the maximum fructose concentration (100 g/L) while MSG is at the moderate concentrations (10 g/L). Conversely, lowest biomass was achieved when both factors are at the minimum level. However, when both factors are at maximum concentration, biomass production was affected negatively with the relative impact of −0.16 (Fig. 2).

This condition could be attributed to the osmotic pressure of the culture medium. When both fructose and MSG were in excess, the relative osmotic pressure of the medium compared to the cellular environment increased. This hyperosmotic pressure may result in an imbalanced of fluid flux which leads to cell shrinkage. In contrast, low concentrations of both fructose and MSG lead to poor carbon and nitrogen supply which caused starvation to the cells^19^. This result is also in agreement with the recent studies conducted by Furlan *et al*.^36^ and Chen *et al*.^37^ which concluded that high nitrogen concentration in the cultivation medium inhibited the growth of *Aurantiochytrium* sp. ATCC PRA-276 and *Aurantiochytrium* sp. respectively.

### Optimization of lipid production

The amount of lipid produced by SW1 varied widely, depending on the parameter and concentration of the studied factors as shown in Table 1. The maximum lipid produced was in standard order 8 (9.23 g/L) and the lowest was in standard order 11 (0.15 g/L) with a maximum to minimum ratio of 61.53. A ratio greater than 10 usually implies that a power transformation is required to increase the normality of the dataset. However, since the normal plot of residuals (Supplementary File 2) confirmed that the dataset follows a normal distribution, therefore the optimization process was carried out without any power or further transformation.

Similar to the response of biomass, the sequential model sum of squares, lack of fit test and R2 value showed that a quadratic equation was the fittest model for regression of the experimental data. ANOVA for the quadratic model was shown in Table 3. These values showed that all the three factors and only the interaction of fructose and agitation speed (AB) gave significant positive effect on lipid production in SW1 (Table 3).

**Table 3:** ANOVA for lipid production by SW1.

Sources	Sum of Squares	DF	Mean Square	F Value	Prob > F
Model	112.67	9	12.52	27.03	<0.0001
A	31.32	1	31.32	67.61	<0.0001
B	23.79	1	23.79	51.35	<0.0001
C	4.14	1	4.14	8.94	0.0136
A^2^	23.94	1	23.94	51.69	<0.0001
B^2^	35.03	1	35.03	75.61	<0.0001
C^2^	0.51	1	0.51	1.11	0.3168
AB	7.32	1	7.32	15.79	0.0026
AC	6.125E-004	1	6.125E-004	1.322E-003	0.9717
BC	1.16	1	1.16	2.51	0.1442
Residual	4.63	10	0.46		
Lack of Fit	2.01	5	0.40	0.76	0.6124
Pure Error	2.63	5	0.53		
Cor Total	117.31	19			
Std. Dev.	0.68		R^2^	0.96	
Adeq Precision	18.27		Adj R^2^	0.91	

p < 0.005 is significant.

Based on the relative impact of the factors (Fig. 2), fructose was shown to give the highest influence on lipid production with the relative impact of 1.90, followed by agitation speed and MSG with the value of 1.30 and 0.76, respectively. Therefore, the experimental values obtained from the central composite design (CCD) were regressed using a quadratic polynomial equation, and the regression equation, expressed in terms of the actual factors, is shown below.

2 $$\begin{array}{rcl}{\rm{Lipid}}\,({\rm{g}}/{\rm{L}}) & = & 3.47239+0.089966\,{\rm{fructose}}+0.044610\,{\rm{agitation}}\,{\rm{speed}}\\  &  & +\,0.055758\,{\rm{MSG}}-\,8.75752{\rm{E}}-004\,{({\rm{fructose}})}^{2}\\  &  & -\,1.23093{\rm{E}}-004\,{({\rm{agitation}}{\rm{speed}})}^{2}-4.54425{\rm{E}}-003\,({\rm{MSG}})\\  &  & +\,2.39062{\rm{E}}-004\,({\rm{fructose}}\times {\rm{agitation}}\,{\rm{speed}})\\  &  & +\,3.12500{\rm{E}}-005\,({\rm{fructose}}\times {\rm{MSG}})\\  &  & +\,5.44643{\rm{E}}-004\,({\rm{MSG}}\times {\rm{agitation}}\,{\rm{speed}})\end{array}$$

#### Effects of the variables on lipid production of SW1

**Figure 4:**
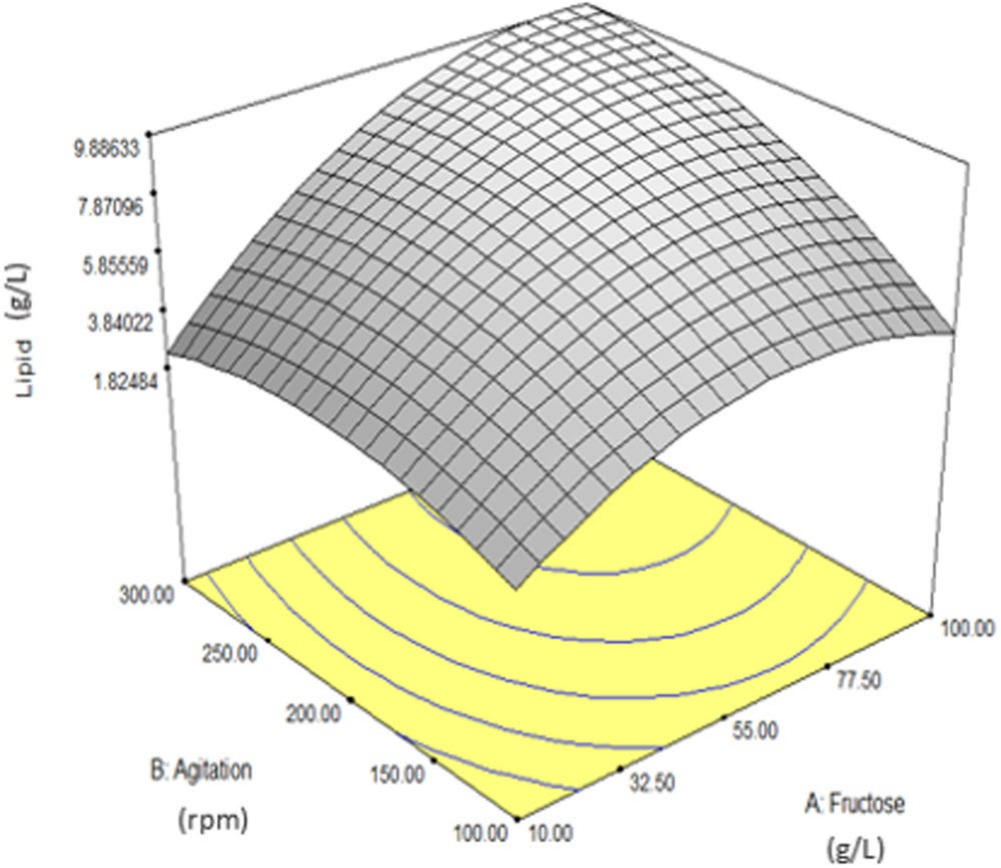
Interaction between fructose concentration and agitation speed (AB) for lipid production of SW1.

As mentioned earlier, only the interaction of fructose and agitation speed (AB) gave significant impact on lipid production (Table 3). Therefore, 3D graph for these factors was plotted and studied (Fig. 4). From the graph, the maximum lipid production was achieved when both factors were at maximum concentration and vice versa. This was due to the fact that both of these factors have a significant impact on the growth of SW1 which consequently resulted in high absolute amount of lipid (g/L). It is reasonable that the factors which favour growth are significant in affecting the lipid yield as the lipid accumulation in SW1is growth-associated as discussed in section 2.1.

However, the results showed that cultivating SW1 with the fructose concentration exceeding 80 g/L gave a negative impact on the lipid production as shown in Fig. 4 as well as in standart order 10 where only 5.02 g/L lipid produced when SW1 was cultivated with 127.5 g/L of fructose in comparison to 7 g/L of lipid produced in standart order 14 to 20 when SW1 was cultivated with 60 g/L fructose (Table 1). This is likely because of the inhibition of cell growth due to hyperosmotic pressure as discussed in Fig. 3(a) which consequently decreased the lipid produced by SW1.

Individual effect of agitation speed (B) showed that optimal lipid production was achieved at the agitation speed of 200 rpm and culturing SW1 beyond this value decrease the lipid production of SW1 (Fig. 4). A study conducted by Zhang *et al*.^38^ reported that a higher agitation speed (between the range of 300–350 rpm) was required to achieved high cell densities but moderate agitation speed (200–250 rpm) was optimal for substantial lipid and DHA accumulation in *Schizochytrium* Sp. FJU-512. Another study conducted by Li *et al*.^39^ who use glycerol as main carbon source however proposed that a rapid, continuous supply of oxygen was required for enhanced lipid production by *Schizochytrium* sp. S3 as the key ezymes responsible for glycerol assimilation is highly dependent on oxygen. Therefore, the utilization of different substrate may lead to different oxygen requirement for *Thraustochytrids* cultivations.

### DHA Optimization

The amount of DHA produced by SW1 ranging from 32.75% to 58.36% of total fatty acids (TFA) with the highest DHA achieved when SW1 was cultivated with 100 g/L fructose, 300 rpm agitation and 16 g/L MSG. The normal plot of data distribution for DHA production was shown in Supplementary File 3. The plot established that the dataset follows a normal distribution and suitable for further optimization.

Similar to the response of growth and lipid produced by SW1, quadratic model was the fittest model for regression of the experimental data. ANOVA for quadratic model was shown in Table 4. These values conclude that only agitation speed (B) and MSG (C) were significant and none of the interaction of factors has the significant positive effect on DHA production by SW1(Table 4). The relative impact of the factors (Fig. 2) on DHA production showed that, MSG, which has the least impact on lipid accumulation gave the maximum influenced in DHA production, followed by agitation speed with the relative impact of 6.56 and 2.79 respectively.

**Table 4:** ANOVA for DHA production by SW1.

Sources	Sum of Squares	DF	Mean Square	F Value	Prob > F
Model	853.35	9	94.82	17.59	<0.0001
A	5.88	1	5.88	1.09	0.3208
B	111.77	1	111.77	20.74	0.0011
C	421.46	1	421.46	78.19	<0.0001
A^2^	0.59	1	0.59	0.11	0.7484
B^2^	219.07	1	219.07	40.64	<0.0001
C^2^	6.45	1	6.45	1.20	0.2997
AB	1.35	1	1.35	0.25	0.6272
AC	1.87	1	1.87	0.35	0.5687
BC	1.47	1	1.47	0.27	0.6128
Residual	53.90	10	5.39		
Lack of Fit	41.27	5	8.25	3.27	0.1100
Pure Error	12.64	5	2.53		
Cor Total	907.26	19			
Std. Dev.	0.79		R^2^	0.98	
Adeq Precision	36.07		Adj R^2^	0.97	

p < 0.005 is significant.

Hence, the experimental values obtained from the central composite design (CCD) were regressed using a quadratic polynomial equation, and the regression equation, expressed in terms of the actual factors, is shown below.

3 $$\begin{array}{rcl}{\rm{DHA}}\,( \% ) & = & 23.48146-0.031384\,({\rm{Fructose}})+0.14050\,({\rm{Agitation}})\\  &  & +\,0.98533\,({\rm{MSG}})+1.37016{\rm{E}}-004\,({{\rm{Fructose}}}^{2})\\  &  & -\,3.07835{\rm{E}}-004\,({{\rm{Agitation}}}^{2})-0.016092\,({{\rm{MSG}}}^{2})\\  &  & +\,1.02812{\rm{E}}-004\,({\rm{Fructose}}\times {\rm{Agitation}})\\  &  & +\,1.72768{\rm{E}}-003\,({\rm{Fructose}}\times {\rm{MSG}})\\  &  & +\,6.12500{\rm{E}}\,004\,({\rm{Agitation}}\times {\rm{MSG}})\end{array}$$

#### Effects of variables on DHA productions by SW1

**Figure 5:**
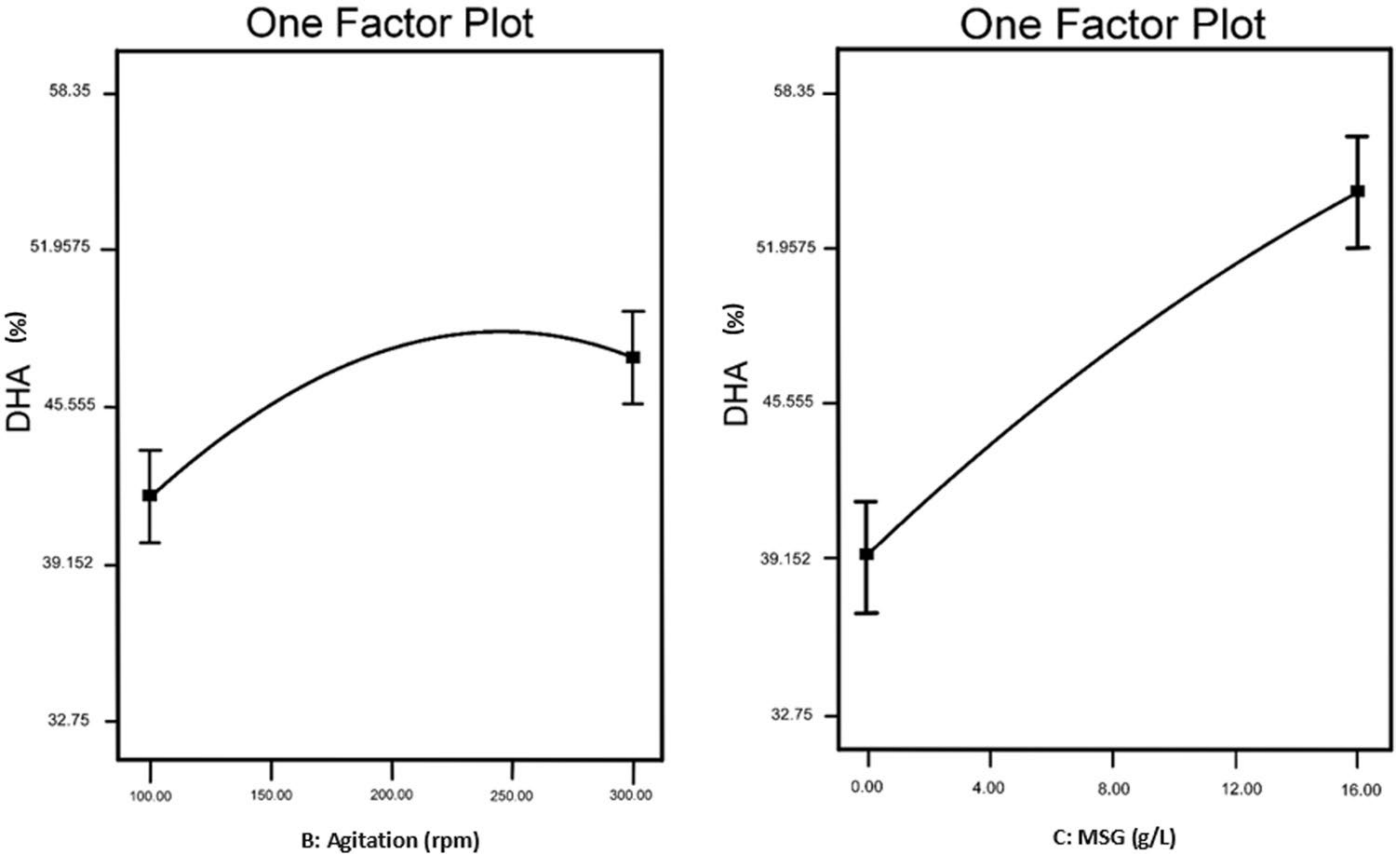
One factor plot of agitation speed and MSG concentration on DHA production by SW1.

As mentioned previously, interactions between factors did not record any significant impact on DHA production (Table 4). However, since the individual effect of agitation speed and MSG showed a positive impact on DHA production, one-factor plot for these two factors was generated (Fig. 5).

Based on this figure, it showed that increasing the MSG concentration from 2 g/L to 16 g/L gave a positive influence on the DHA production by SW1. The role played by MSG in enhancing DHA production was also consistent with studies reported by Burja *et al*.^40^, which showed that, supply of 10 g/L MSG to culture *Traustochytrium* sp. ONC-T18 led to an increase in lipid DHA content of 43%. Moreover, Ren *et al*.^10^ have also noted that MSG has a positive effect on the accumulation of DHA in the *Aurantiochytrium* sp. This suggests that MSG is the favourable nitrogen source in enhancing DHA production by *Thraustochytrids*.

However, result showed that the optimal agitation speed for maximum DHA production was 250 rpm and increasing the agitation speed beyond this value decreases the amount of DHA produced by SW1. This is most probably due to inhibition of DHA synthesis as increasing the agitation speed could increase the dissolved oxygen in the medium^22^. The favourable result of increasing the agitation on the production of biomass and DHA was also reported by Song *et al*.^41^ who observed that increasing agitation speeds to 250 rpm improved biomass and DHA production of *Schizochytrium limacinum* OUC88 to maximum concentrations of 24.1 g/L and 4.7 g/L, respectively. The positive effect of agitation speed could be related to the fact that higher agitation speed resulted in better mixing of medium with improved culture homogeneity as well as improved transfer of oxygen and nutrients, which in turn increased microbial cell growth and subsequent biomass and DHA production^16^.

Based on the regression analysis of the model equation for biomass and DHA production of SW1, the optimum levels of the variables were estimated. The optimum conditions were 70 g/L fructose, 250 rpm agitation speed and 10 g/L MSG. The predicted optimal biomass, lipid and DHA production were 19.50 g/L, 9.4 g/L and 50.6% respectively according to the model equation (Eqs 2, 3 and 4) generated by the software.

### Model Validation

Further experiments were carried out to validate the predicted biomass, lipid and DHA production of SW1 under the estimated optimum conditions generated by the software. The data for biomass, lipid, DHA concentration and productivity as well as the percentage of increment for each response prior and after the optimization were shown in Table 5.

**Table 5:** Comparison of biomass, lipid, DHA concentration and productivity as well as percentage of increment prior and after the optimization.

Parameter	Unit	Before optimization	After optimization	Percentage of increment
Biomass	g/L	14.80 ± 1.2	19.00 ± 1.99	28.40 ± 3.90
Lipid	g/L	6.70 ± 0.25	9.13 ± 0.15	36.27 ± 2.85
DHA	g/L	3.50 ± 0.16	4.75 ± 0.26	35.70 ± 3.03
DHA productivity (Y/t)	g/L/day	0.88 ± 0.04	1.20 ± 0.07	36.36 ± 3.73

The biomass, lipid and DHA production obtained from optimized conditions were 19 g/L, 9.13 g/L and 52% respectively, with absolute DHA content achieving 4.75 g/L which is comparable to the predicted values and significantly higher (p < 0.05; Supplementary File 4) than the original medium prior to the optimization (Table 5). Therefore, this experiment validated the model and the RSM-CCD can be used as an optimization tool to enhance DHA production in SW1. The optimized biomass and DHA production by SW1 was higher than previously reported in *Thraustochytrium aureum*, *Thraustochytrium roseum* and *Schizochytrium magrovei* g-13 which were cultured in shake flask under optimal conditions for 5–12 days^10,42,43^. Furthermore, the growth and DHA produced by SW1 was superior to recently reported study by Sahin *et al*.^44^ which cultivated *Schizochytrium* sp. S31 in media supplemented with fructose as sole carbon sources. In addition, the absolute amount of DHA (g/L) produced by SWI (this study) is also equivalent to commercial *Thraustochytrids* strain such as *Aurantiochytrium limacinum* OUC88, *Schizochytrium* sp. KH 105, *Schizochytrium* sp. SR21 and *Aurantiochytrium* sp. KRS101, indicating that it has a potential to be developed as an industrial strain^27,41,45,46^.

### Fermentation Scale-Up

**Figure 6:**
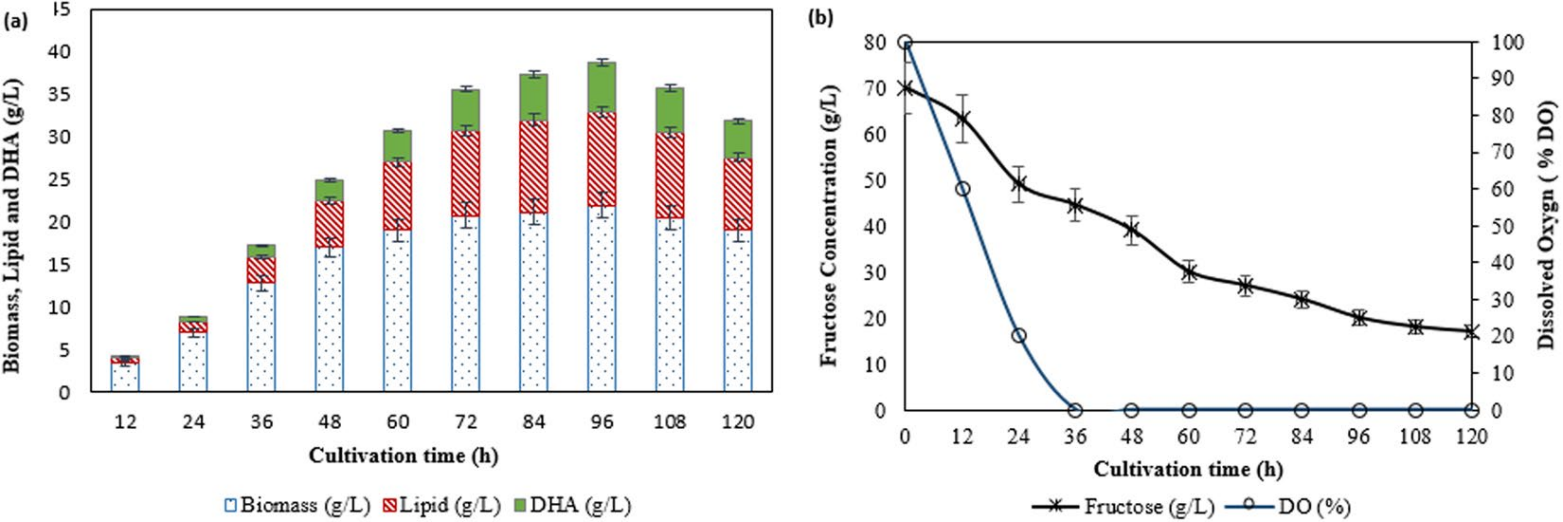
(**a**) Growth, lipid and DHA yield (g/L) and as well as (**b**) fructose assimilation (g/L) and dissolved oxygen (%) of *Aurantiochytrium* SW1 grown in 5 L fermenter at 30 °C, agitation speed of 250 rpm and aeration rate of 1 vvm for 120 h.

In order to establish the reproducibility of the estimated optimum condition data in large-scale productions, an upscaling experiment was conducted in 5 L benchtop fermenter with 3 L working volumes (Fig. 6). The growth, lipid and DHA pattern of SW1 was comparable with the study reported by Yu *et al*.^25^, which was grown in the media supplemented with high fructose corn syrup (HFCS) as sole carbon sources at a similar cultivation scale (5 L). The result showed that SW1 produced up to 21.8 g/L biomass, 10.0 g/L lipid and 5.0 g/L DHA which was slightly higher compared to the production in shake flask (Table 5). This might be attributed to a better agitation and oxygen supply which thereby increased the growth and DHA production by SW1. Therefore, this data confirmed the reproducibility and applicability of the shake flask data at 5 L scale. The data obtained from this study could be a bridge towards evaluating the idea on industrial scale productions.

## Materials and Methods

### Organism and Culture Conditions

The *Thraustochytrids* used in this study is *Aurantiochytrium* SW1 (GenBank: KF500513), provided by Microbial Physiology Lab, *School of Biosciences and Biotechnology*, *Universiti Kebangsaan Malaysia* and has been deposited in UNiCC UPM under the accession number of [UPMC 963]. This organism was maintained on seawater nutrient agar (SNA) as slant culture which contained 28 g/L nutrient agar and 17.5 g/L artificial seawater accounting for 50% salinity. Seed cultures were prepared by inoculating 100 mL of a seeding broth with a strip of SNA slant agar containing approximately ten colonies of 48 h old of SW1 cells in 500 mL Erlenmeyer flasks. Seed cultures were then incubated at 30 °C for 48 h under an agitation rate of 200 rpm. The medium used in seed cultures contained 60 g/L fructose, 2 g/L yeast extract, 8 g/L monosodium glutamate (MSG) and 6 g/L artificial sea salt. The composition of sea salt used in this study was described by Manikan *et al*.^47^. Seed culture with an inoculums size of 10% (v/v) was inoculated into 100 mL production medium containing similar medium composition used for seed culture but fructose, agitation speed and MSG were set according to the levels shown in the experimental design (Table 1). The cultures were incubated for 96 hours at 30 °C.

### Experimental Design

Twenty sets of experiment were designed and analyzed using central composite design (CCD - response surface; Design Expert Software (DOE; version 6.0.10, Stat-Ease, USA) as shown in Table 1. Three factors which are concentration of fructose, agitation speed and concentration of MSG were chosen to be optimized for DHA production. The chosen ranges were: fructose 20–100 g/L, agitation speed 100–300 rpm and MSG 2–16 g/L.

Statistical analysis using ANOVA with the DOE software was used to estimate the optimal culture conditions for maximum DHA production by SW1. The coefficients in the second-order polynomial (Eq. 1) were calculated by multiple regression analysis of the experimentally obtained results.

4 $$Y={b^{\prime} }_{0}+\sum _{i=1}^{n}{b}_{i}{X}_{i}+\sum _{i=1}^{n}{b}_{ii}{X}_{i}^{2}+\,\sum _{i=1}^{n}\cdot \sum _{j\ge 1}^{n}{b}_{ij}{X}_{i}{X}_{j}$$

where *Y* is the predicted response, *b′*_0_ is the constant coefficient, *bi* is the linear coefficient, *b*_*ij*_ is the interaction coefficient, *b*_*ii*_ is the quadratic coefficient, and *X*_*i*_ and *X*_*j*_ are coded values.

### Determination of Fructose Concentration

Fructose concentration was measured by HPLC (12000 Series, Agilent Technologies, Palo Alto, CA, USA) using a Shodex Asahipak NH2P-50 4E column (4.6 mm ID × 250 mm; Shodex, Kanagawa, Japan). The fructose were detected with a refractive index detector (RID; 1200, Agilent Technologies, Palo Alto, CA, USA) at 30 °C with a flow rate 1 mL/min of a mixture of acetonitrile (J.T. Baker Chemical Co. Phillipsburg, NJ, USA) and water (H2O/CH3CN = 40/60) as a mobile phase.

### Determination of Dry Cell Weight

*Aurantiochytrium* SW1 cells were harvested by centrifugation method at 4000 × g for 10 min using Eppendorf centrifuge 5810 R. The cells were then washed twice using 25 mL of sterile distilled water. Cell samples were then oven-dried at 80 °C for 24 h and weighed. Biomass obtained was expressed as gram dried cell per litre of growth medium.

### Lipid Extraction and Fatty Acid Analysis

Lipid extraction was performed using chloroform-methanol (2:1, v/v), as described by Folch *et al*.^48^. The extract was vaporized at room temperature and dried in a vacuum desiccator until constant weight was attained. Fatty acid compositions of the samples were determined as fatty acid methyl esters (FAMEs) by gas chromatography (HP 5890) equipped with a capillary column (BPX 70, 30 cm, 0.32 µm). FAME was prepared by dissolving 0.05 g of the sample in 0.95 mL hexane, and the mixture was added to 0.05 ml of 1 M sodium methoxide. The injector was maintained at 200 °C. Then, 1 µl of sample was injected using helium as a carrier gas with a flow rate of 40 cm^3^ min^−1^. The temperature of the GC column was gradually increased at 7 °C min^−1^ from 50 (5 min hold) to 200 °C (10 min hold). Fatty acids peaks were identified using Chrome Leon chromatography software (Dionex, Sunnyvale, California, USA). FAMEs were identified and quantified by comparison with the retention time and peak areas of SUPELCO (Bellefonte, PA, USA).

### Cultivation in 5L Fermenter

An up-scaling experiment was carried out in a 5L bench-top bioreactor (Minifors-Infors HT) with a working volume of 3L to validate the reproducibility of the data obtained in shake flask fermentation. The culture temperature was controlled at 30 °C and the impeller speed was fixed at 250 rpm. The aeration was controlled at 1 vvm. Samples were collected every 12 h for 120 h to determine the biomass, lipid and DHA production by SW1.

### Statistical analysis

All the experiments were conducted in three replicates. The effect of studied factors in Table 5 was analyzed statistically by one-way analysis of variance (ANOVA) using SPSS software (SPSS Inc., Version 16.0) with the significance level set at 5%.

## Electronic supplementary material

Supplementary files
