# Supplementary material for: Optimization of Culture Conditions for Enhanced Growth, Lipid and Docosahexaenoic Acid (DHA) Production of Aurantiochytrium SW1 by Response Surface Methodology
Source: Sci Rep. 2018 Jun 11;8:8909. doi: 10.1038/s41598-018-27309-0 (PMC5995909; doi:10.1038/s41598-018-27309-0)
Supplement: Supplementary file 1 — Supplementary files [file 41598_2018_27309_MOESM1_ESM.docx]

**Supplementary files**

**OPTIMIZATION OF CULTURE CONDITIONS FOR ENHANCED GROWTH, LIPID AND DOCOSAHEXAENOIC ACID (DHA) PRODUCTION OF *Aurantiochytrium* SW1 BY RESPONSE SURFACE METHODOLOGY**

Yusuf Nazir^1, 3^, Shuwahida Shuib^1^, Mohd Sahaid Kalil^2^, Yuanda Song^3*^ & Aidil Abdul Hamid^1*^

^1^School of Biosciences and Biotechnology, Faculty of Science and Technology, Universiti Kebangsaan Malaysia

^2^Department of Chemical and Process Engineering, Faculty of Engineering and Built Environment, Universiti Kebangsaan Malaysia

^3^ Colin Ratledge Center for Microbial Lipids, School of Agriculture Engineering and Food Science,

Shandong University of Technology, Zibo 255049, China

* Correspondence authors: Yuanda Song & Aidil Abdul Hamid

Email: [ysong@sdut.edu.cn](mailto:ysong@sdut.edu.cn) (Yuanda Song), [aidilmikrob@gmail.com](mailto:aidilmikrob@gmail.com) (Aidil Abdul Hamid)

**Supplementary file 1**

**Normal plot of residual for biomass data**

**Supplementary file 2**

**Normal plot of residual for lipid data**

**Supplementary file 3**

**Normal plot of residual for DHA data**

**Supplementary file 4**

Results of one-way ANOVA for the comparison of biomass, lipid, DHA concentration and productivity as well as percentage of increment prior and after the optimization.

| **Biomass (g/L)** | | | | | |
| --- | --- | --- | --- | --- | --- |
| Source of Variation | *SS* | *df* | *MS* | *F* | *P-value* |
| Between Groups (before and after optimization) | 26.46 | 1 | 26.46 | 9.7458 | 0.0354 |
| Within Groups | 10.86 | 4 | 2.715 |  |  |
|  |  |  |  |  |  |
| Total | 37.32 | 5 |  |  |  |
| **Lipid (g/L)** | | | | | |
| Source of Variation | *SS* | *df* | *MS* | *F* | *P-value* |
| Between Groups (before and after optimization) | 8.85735 | 1 | 8.85735 | 215.2454 | 0.00012 |
| Within Groups | 0.1646 | 4 | 0.04115 |  |  |
|  |  |  |  |  |  |
| Total | 9.02195 | 5 |  |  |  |
| **DHA (g/L)** | | | | | |
| Source of Variation | *SS* | *df* | *MS* | *F* | *P-value* |
| Between Groups (before and after optimization) | 2.34375 | 1 | 2.34375 | 51.3417 | 0.002008 |
| Within Groups | 0.1826 | 4 | 0.04565 |  |  |
|  |  |  |  |  |  |
| Total | 2.52635 | 5 |  |  |  |
| **DHA productivity (g/L/day)** | | | | | |
| Source of Variation | *SS* | *df* | *MS* | *F* | *P-value* |
| Between Groups (before and after optimization) | 0.15041 | 1 | 0.15041 | 52.470 | 0.001927 |
| Within Groups | 0.01146 | 4 | 0.00286 |  |  |
|  |  |  |  |  |  |
| Total | 0.16188 | 5 |  |  |  |

P<0.05 is significant

**Table Legends**

Supplementary file 4: Results of one-way ANOVA for the comparison of biomass, lipid, DHA concentration and productivity as well as percentage of increment prior and after the optimization.

**Figure Legends**

Supplementary file 1: Normal plot of residual for biomass data

Supplementary file 2: Normal plot of residual for lipid data

Supplementary file 3: Normal plot of residual for DHA data
